# Fabrication of Iron Oxide/Zinc Oxide Nanocomposite Using Creeper *Blepharis maderaspatensis* Extract and Their Antimicrobial Activity

**DOI:** 10.3389/fbioe.2020.595161

**Published:** 2020-12-18

**Authors:** Heba Salah Abbas, Akilandeswari Krishnan, Muddukrishnaiah Kotakonda

**Affiliations:** ^1^National Organization for Drug Control and Research, Giza, Egypt; ^2^Scientist Under Scheme of Asian Research Training Fellowship-Developing Countries Scientist (RTF-DCS), Federation of Indian Chambers of Commerce and Industry (FICCI), New Delhi, India; ^3^Department of Pharmaceutical Technology, Bharathidasan Institute of Technology, Anna University, Tiruchirappalli, India

**Keywords:** iron oxide/ZnO nanocomposite, *Blepharis maderaspantensis*, antimicrobial activity, characterization, magnetic nanosystem

## Abstract

Green nanotechnology has recently had a significant influence on advances in biological applications. The surface manipulation of iron oxide NPs by zinc oxide is increasing attention for biomedical research. Therefore, this work focused on the phytochemicals of creeper *Blepharis maderaspantensis* (BM) water extract for synthesizing iron oxide NPs and iron oxide/zinc oxide nanocomposite. The UV spectrum analysis showed a wavelength redshift from 294 to 302 nm of iron oxide/ZnO nanocomposite, and the polydispersity index revealed that the perfect preparations of iron oxide NPs were prepared by boiling 0.25 g of the plant in deionized water then the filtrate added to ferric chloride (1:1 v/v). The HRTEM results also illustrated that amorphous iron oxide NPs are spherical and irregular in shape. However, the iron oxide/ZnO nanocomposite showed a rod shape of ZnO with an average length and width of ∼19.25 ± 3.2 × 3.3 ± 0.6 nm surrounding amorphous iron oxide NPs. Furthermore, a high antimicrobial activity with MRSA and *E. coli* was demonstrated by iron oxide NPs. However, because of instability and negative surface charge of the iron oxide nanocomposite, there was no antimicrobial activity. Future cytotoxic studies of the iron oxide NPs synthesized with polyphenols of BM extract are desirable, and their applications in medical purposes will be recommended.

## Introduction

In developing countries, nanotechnology is being used to treat microbial infections. The paraglider term for this type of nanotechnology is nanomedicine (Freitas, 1999). Current issues involve the development and use of nanomaterials and their possible adverse effects on the environment. The use of toxic chemicals for the reducing process or as a capping agent produces hazardous products in the environment during nanomaterial synthesis. The use of toxic chemical compounds during nanomaterial synthesis for the reduction process or as capping agent generates hazard products in the environment. To save our environment, it could be a good starting point for nanomaterial research without the presence of toxic elements, thus heading toward green nanotechnology, concentrating on materials and methods free from undesirable side effects on the environment (de la Guardia, 2014). Through interfacing them with biological molecules or structures, functionalities can be applied to nanomaterials; thus, for *in vivo* and *in vitro* biomedical purposes, nanomaterials can be useful.

Plant phytochemicals are employed for the synthesis of nanoparticles as green methods due to their reducing properties for metal compounds into nanometals (Prathna et al., 2010). The creeper *Blepharis maderaspatensis* (BM), which has been used as a traditional medicine for treating headache, bone fracture, diarrhea suppression, and lactate induction, has been one of the most important plants rich in phytochemicals, such as phenols, carbohydrate, flavonoid, and protein (Baskar et al., 2012; Abimbola et al., 2013).

Iron oxide NPs are vital metal oxide nanoparticles that can be used in several therapeutic applications (Pankhurst et al., 2003; Sharaf et al., 2019). Surface manipulation is necessary to make iron oxide NPs biocompatible. Antimicrobial activity is primarily aimed at developing nanoparticles by altering the surface of nanoparticles, with no impact on healthy cells (Toropova et al., 2017). The low reactivity, agglomeration, and oxidation of iron oxide NPs can be avoided by coating with another metallic oxide NPs, such as zinc NPs (Gupta and Gupta, 2005; Sun et al., 2014; Bisht et al., 2016). The surface coating of iron oxide NPs not only decreases the cytotoxicity of iron oxide NPs but also increases the stability and efficiency of antimicrobial potential of Iron oxide NPs (Abbas and Krishnan, 2020).

In the present work, we have attempted to describe the green synthesis of iron oxide NPs and their coating by ZnO NPs using creeper *Blepharis maderaspatensis* extract in ambient temperature for increasing the reactivity of iron oxide NPs as antimicrobials and to prevent their agglomeration and oxidation; this is a first ever study that reported the synthesis of iron oxide NPs and their coating by ZnO NPs using creeper *Blepharis maderaspatensis* obtained from Kanyakumari District of Tamil Nadu, India.

## Materials and Methods

### Chemicals

The chemicals used were anhydrous ferric chloride (98%), zinc nitrate hexahydrate (99%), sodium hydroxide, potato dextrose agar (PDA), and Muller–Hinton agar (MHA) were purchased from Himedia and SD Fine Chemicals, Mumbai, India; methicillin-resistant *Staphylococcus aureus* (MRSA), *E. coli*, *Pseudomonas aeruginosa*, and *Candida albicans* were received from KAP Viswanathan Medical College, Tiruchirappalli, India.

### Collection of *Blepharis maderaspatensis* (BM)

The plant was collected in the Kanyakumari District of Tamil Nadu, India from the natural environment. Botanist Dr. V. Chelladurai, Research Officer-Botany (Retd.), certified the plant. At the herbarium of the Entomology Research Institute, Loyola College, Chennai (India), the Central Research Council in Ayurveda and Siddha, the government of India, and the herbarium of India, the plant was carefully examined and botanically identified as BM of the Acanthaceae family. In flowing tap water, the samples are thoroughly washed to extract soil particles and adhered debris and washed with clean distilled water at last. In a mechanical grinder, the specimen was dried and pulverized, then it passed through a 40-mesh sieve. The powdered plant material was then packed in an airtight polythene bag until use (Souad et al., 2010).

### Preparation of BM Extract

Different weights of *BM* powder (0.25, 0.5, 1, and 2 g) were soaked in 100 ml Milli-Q water or boiled at 80°C for extraction. Then, samples were filtered and stored at 20°C before use. After cooling, the samples were filtered and stored at 20°C before use.

### Preliminary Screening of Phytochemicals in BM Extract

The aqueous extract of BM was screened for the presence of phytochemicals, which reflect the bioactive substances in the extract, such as carbohydrates, phenols, glycosides, proteins, and alkaloids which were screened (Habbal et al., 2011).

### Biological Synthesis of Iron Oxide NPs

Iron oxide NPs were designed by adding 0.2 M ferric chloride to *Blepharis* extract in a 1:1 volume ratio. The mixture was shaken for 1 h and allowed to stand at ambient temperature for half an hour (Mahdavi et al., 2013). Iron oxide NPs were designed by adding 0.2 M ferric chloride to *Blepharis* extract in a 1:1 volume ratio (Das et al., 2016). Iron oxide nanosuspension was characterized by using a UV spectroscope and Zetasizer.

### Synthesis of Iron Oxide @ ZnO NPs

According to section “Preparation of BM extract,” the appropriate biologically synthesized iron oxide nanosuspension was selected for coating by ZnO NPs using the colloidal technique. One ml of sodium zincate solution was diluted and added to 100 ml of biologically synthesized iron oxide nanosuspension at 95 ± 2°C under vigorous stirring for 30 min (CLSI, 2004).

### Characterization of Nanomaterials

Proper iron oxide NPs and coating with ZnO NPs were characterized by using various instruments, such as UV-visible spectroscope (Shimadzu UV-2600), high-resolution transmission electron microscope (HRTEM, JEOL-JEM-2010), dynamic light scattering (DLS) instrument, X-ray diffractometer (X’Pert3 powder X-ray Diffractometer, PANalytical), and Fourier transform infrared spectroscope (FTIR) (JASCO 4600).

### Antimicrobial Studies Using Agar Disc Diffusion Method

Antimicrobial activity of iron oxide NPs, coating with ZnO NPs, plant water-soaked extracts, and plant water-boiled extracts was investigated against methicillin-resistant *Staphylococcus aureus* and *Escherichia coli* by using the agar disc diffusion method according to the CLSI, 2012 and Balouiri et al. (2016). The Muller–Hinton agar (MHA) plates were inoculated with 0.5 McFarland turbidity of bacterial cultures. The sterile discs (6 mm) were moistened with nanosuspensions under aseptic conditions and stored in the refrigerator for soaking. The tetracycline disc was placed on the surface of the agar and incubated in an incubator for 24 hrs. The temperature of the pathogenic bacteria was maintained at 37°C, and the diameter of the inhibition zone was measured by using the Himedia zone reader (Anouar et al., 2012).

## Results and Discussion

**FIGURE 1:**
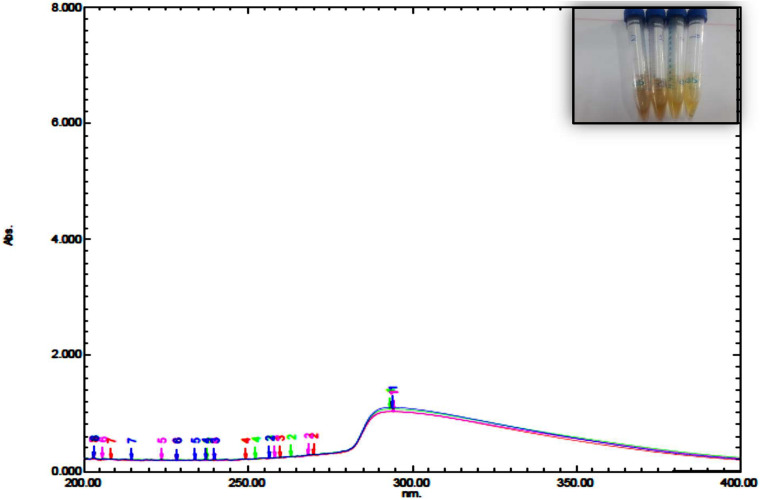
UV spectrum of iron oxide NPs synthesized by *Blepharis maderaspatensis* water-soaked extract.

**FIGURE 2:**
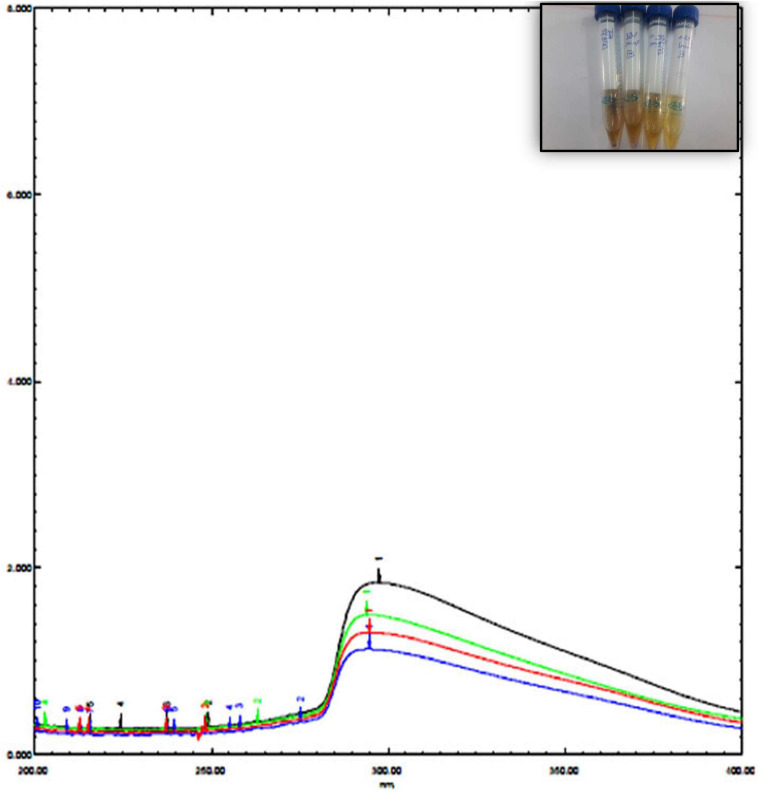
UV spectrum of iron oxide NPs synthesized by *Blepharis maderaspatensis* boiled-water extract.

The UV spectrum of blepharis aqueous extract and synthesized iron oxide NPs was analyzed by using a UV-visible spectrophotometer as shown in Figure 1. Different absorption peaks indicated the enrichment of plant extracts with polyphenols as silybin or aromadendrin or trans-2S or 3S-taxifolin, naringenin, or genistein, and catechin (2S,3R) or epicatechin (2R,3R) and isoluteolin (Huang et al., 2015). Formation of iron oxide NPs due to combinations of various polyphenol substances in *Blepharis* aqueous extract induced the reduction process. The absorbance peaks at 296 nm confirmed the production of iron oxide nanoparticles and also peak at 272.8 due to the capping of polyphenols, which allowed the stabilization of these nanoparticles (Njagi et al., 2011; Vemula et al., 2013). The characteristic absorption bands of monodispersed iron oxide nanoparticles ranged from 290 to 300 nm (Das et al., 2014). The result of the iron oxide NP absorption peak agreed with those obtained by Das et al. (2014), Muthukumar and Matheswaran (2015), and Abbas et al. (2020b). The BM extract was prepared by using different weights, which varied from 0.25, 0.5, 1, to 2 g. Using a soaking extraction method, it showed no distinctive differences in the absorption peaks (around 294 nm) by using different weights of the plant (Figure 1). However, by using the boiling extraction method, there is a tin redshift from 294 to around 296, and 298 nm when the weight of the plant increased from 0.25 g to 1, and 2 gm, respectively (Figure 2). With increasing plant extract concentration, consequent color changes were observed from yellowish-brown to dark yellowish-brown and black for iron oxide nanoparticulates. In that concern, Dwivedi and Gopal (2010) proved that the concentrations of the plant extract and their biological constituents are recognized to influence on the nanoparticles production (Dwivedi and Gopal, 2010).

According to screening for DLS data, the preferred preparations for the synthesis of iron oxide NPs was by using boiled 0.25 g BM extract then the filtrate was added to ferric chloride (1:1 v/v). This preparation of iron oxide NPs gives 48 and 52% of the sample with a small diameter of particle size 19.26 ± 3.079 nm and 133.5 ± 17.8 nm, respectively (Table 1). Furthermore, the PDI value was 0.7 which indicates the stability of the colloidal suspension. Danaei et al. (2018) explained when PDI standards greater than 0.7 indicate the instability and wide particle size distribution. In this case, the colloidal suspension is improper for analyzing by DLS, and this is what happened when other weights of plants were used, as shown in Table 1.

**TABLE 1:** Effect of plant weight and method of extraction on the size of nanoparticles, intensity, and polydispersity index.

Weight of plant (g)	Method of extraction	Size	Intensity (%)	Polydispersity index (PDI)
0.25	Soaking	140.9 ± 14.54	71.9	0.936
		7.79 ± 0.961	28.1	
	Boiling	133.5 ± 17.8	52	0.7
		19.26 ± 3.079	48	
0.5	Soaking	276.5 ± 27.96	100	0.612
	Boiling	484.4 ± 205.5	60.6	1
		4328 ± 961.8	22.7	
		19.88 ± 6.410	16.7	
1	Soaking	77.96 ± 5.679	100	1
	Boiling	105.7 ± 7.5	83.1	1
		7.4 ± 0.6	16.9	
2	Soaking	1556 ± 1233	100	0.430
	Boiling	–	–	–
5	Soaking	205.5 ± 15.02	100	0.746

**FIGURE 3:**
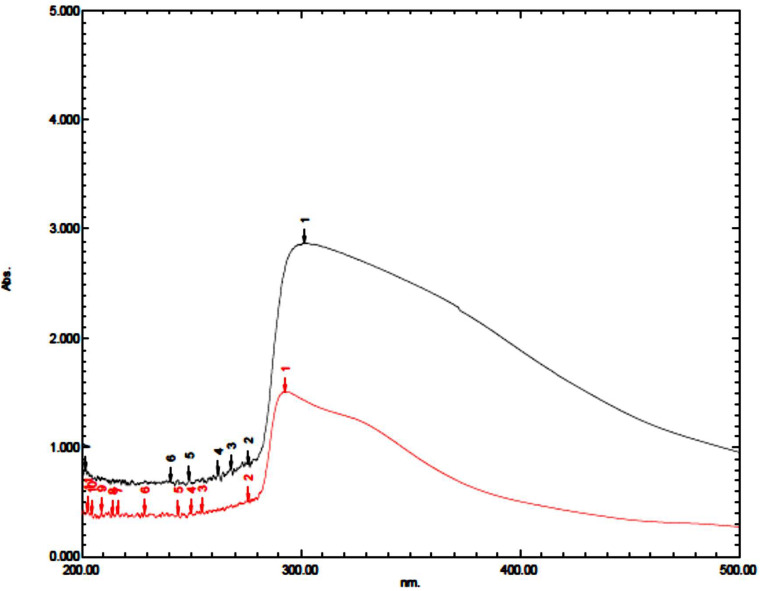
UV spectrum of iron oxide/ZnO nanocomposite (black curve) synthesized by *Blepharis maderaspatensis* boiled-water extract (red curve).

**FIGURE 4:**
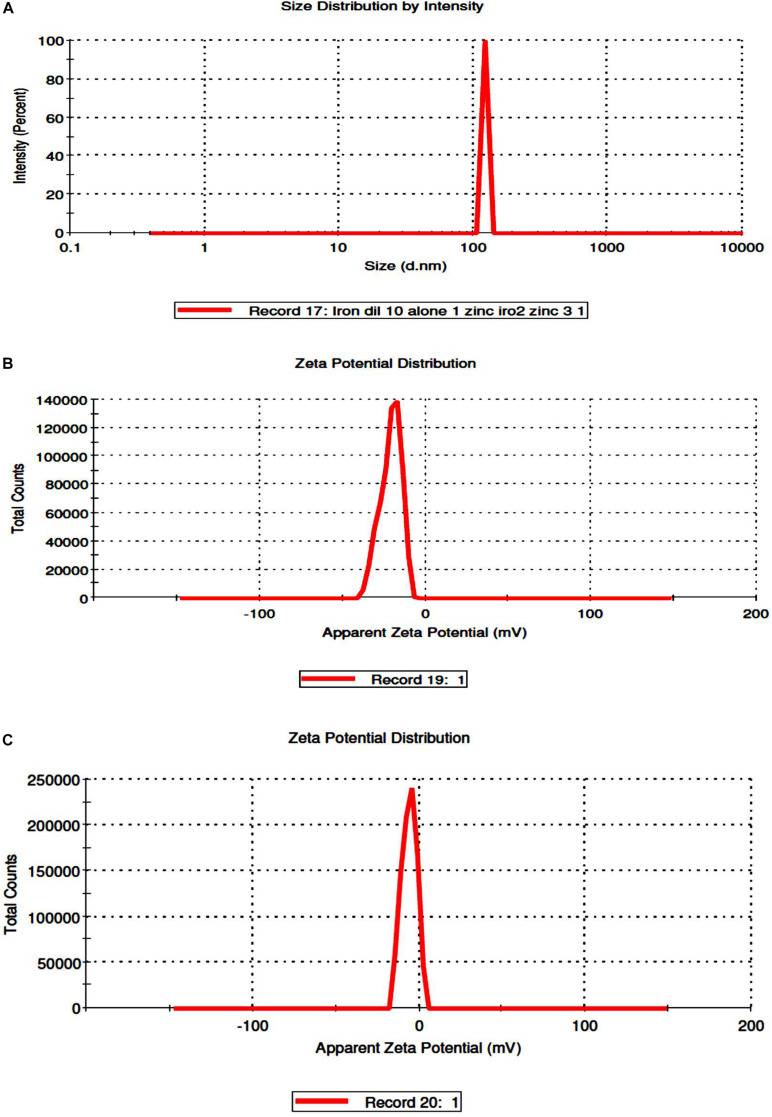
Determination of size distribution iron oxide/zinc oxide nanocomposite **(A)** and zeta potential of uncoated iron oxide NPs and iron oxide/zinc oxide nanocomposite, respectively **(B,C)** by using dynamic light scattering.

Coating of iron oxide NPs biosynthesized by BM extract (0.25 gm weight of BM) was recognized by UV spectroscopy. Subsequent color change takes place from yellowish-brown to brownish orange color where the orange color is characteristic for ZnO NPs. Redshift for the wavelength from 294 nm to 302 nm occurs after coating iron oxide NPs by ZnO NPs (Figure 3). There is no distinctive SPR difference between both iron oxide NPs and ZnO NPs. Hence, the absorption peaks are close to each other. However, in other literature, the coating of AgNPs with ZnO NPs causes a blueshift for the SPR due to distinctive SPR differences between both AgNPs and ZnO NPs (Das et al., 2016; Abhilash et al., 2019). The mean average diameter of ZnO-coated iron oxide NPs was 122.4 nm using DLS, but the PDI was 1 which reflects a broad nanoparticle size distribution and instability (Figure 4). Furthermore, the zeta potential for iron oxide NPs and iron oxide/ZnO nanocomposite was -20.9 ± 6.24 mV and -6 ± 4.43 mV, respectively (Figure 4). Similar results of the negative zeta potential were obtained in the case of preparation of the Fe_2_O_3_/CuO nanocomposite by Abhilash et al. (2019). However, positive zeta potential results were obtained in the preparation of α the Fe_2_O_3_/ZnO nanocomposite (24.7 ± 5.26 mV) by Abbas et al. (2020b).

**FIGURE 5:**
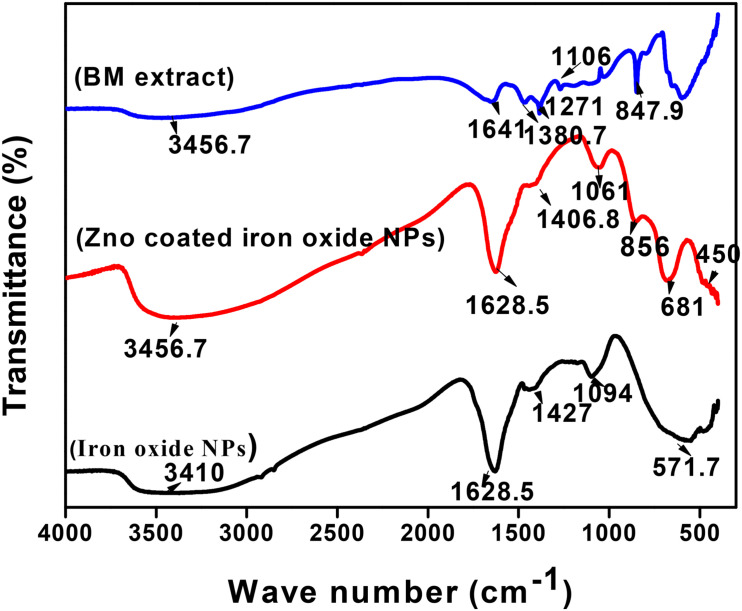
FTIR spectroscopy of iron oxide NPs (black curve), iron oxide/ZnO nanocomposite (red curve), and *Blepharis maderaspatensis* boiled-water extract (blue curve).

The mechanism non-coated and coated iron oxide NP biosynthesis depends on biologically active components of the BM plant, such as polyphenols, proteins, and reducing sugars (Table 2). IR spectra of dried BM revealed various functional groups of these components as follows: 3,456.7 cm^–1^ peak corresponds to O–H stretching vibration of phenols, 1,641 cm^–1^ is assigned to –C = C– stretching vibration of alkene, 1,380.7 cm^–1^ represents the nitrogenous compound, 1,271 cm^–1^ peaks indicated the presence of C–O stretching vibration of carboxylic acid esters, 1,106 cm^–1^ is due to existence of C–N aliphatic amines, and 847.9 cm^–1^ corresponds to N–H wag stretching vibration of primary or secondary amines. To elucidate the functional group responsible for the biosynthesis of non-coated and coated iron oxide NPs, their IR was compared with the dried BM IR spectrum (Figure 5). Our IR results for non-coated and coated iron oxide NPs showed that an intensive absorption spectrum for O–H was observed at 3,410 cm^–1^, –C = C– of alkene was assigned to 1,628.5 cm^–1^, C–C stretching vibration was observed at 1,427, 1,406 cm^–1^, C–O corresponds to 1,094, 1,061 cm^–1^, and a strong absorption band was observed at 571.7 cm^–1^ assigning Fe–O vibration which elucidates the formation of iron oxide NPs, which totally agreed with Waldron (1955). However, in the case of ZnO-coated iron oxide NPs, characteristic absorption bands of Zn–O and Fe–O are noticed at 450 and 681 cm^–1^, respectively, which were also proved by Anžlovar et al. (2012) and Mahamuni et al. (2019). The increase of the wavelength of Fe–O is due to bond breakage and rearrangement of electrons (Shahwan et al., 2011). According to the overall observations, IR spectroscopy could indicate that polyphenols, proteins, and reducing sugar characters over the surface of iron oxide NPs were the cause of synthesis and stabilization of iron oxide NPs as reported by previous studies by Abbas et al. (2020a, b).

**TABLE 2:** Phytochemical analysis of *Blepharis maderaspatensis* aqueous extract and iron oxide NPs.

Name of test	BM aqueous extract	Iron oxide NPs
Test of alkaloids (Mayer’s reagents)	–	–
Protein test (ninhydrin test)	+	+
Test of glycosides (Legal’s test)	–	–
Test of carbohydrate (Molish test)	++	+
Phenol test	+++	+++

**FIGURE 6:**
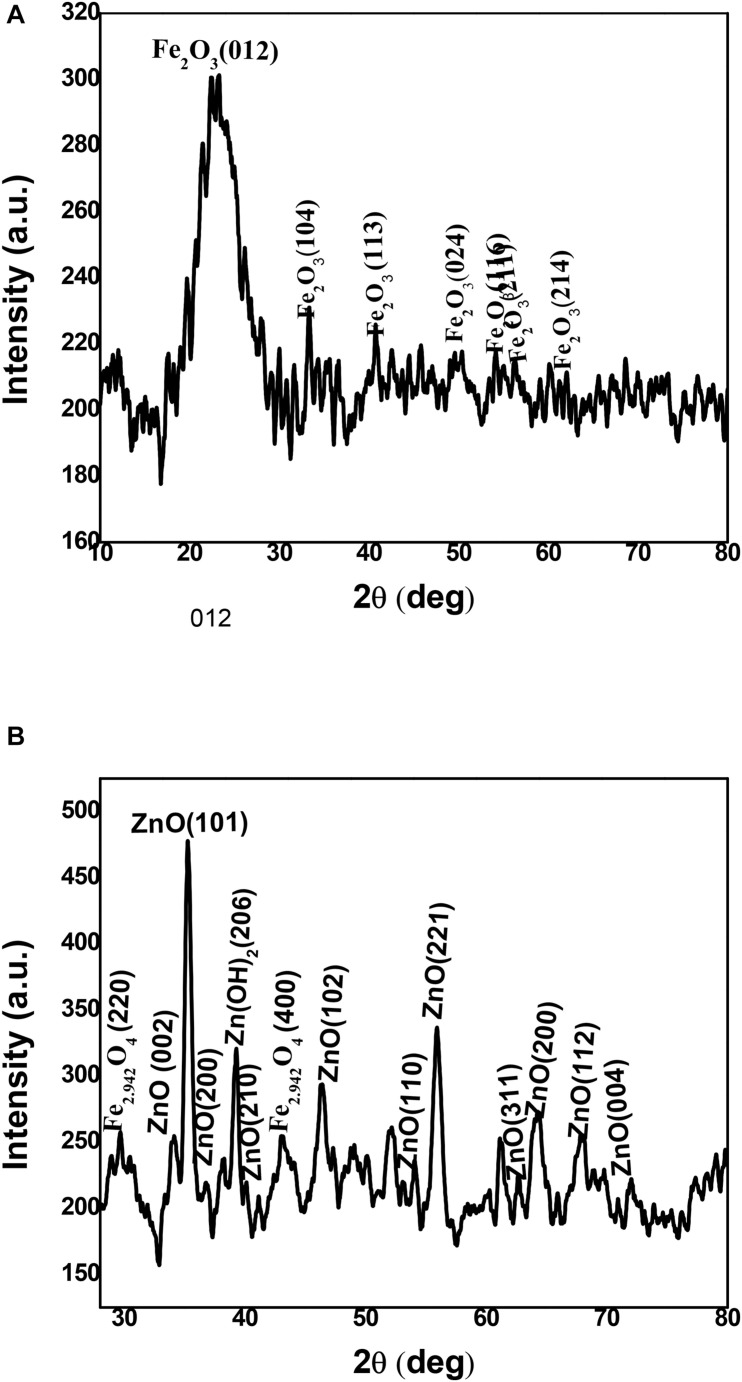
XRD spectroscopy of iron oxide NPs **(A)** and iron oxide/ZnO nanocomposite **(B)**.

The physical phase of non-coated and coated iron oxide NPs was examined by using XRD as in Figure 6. The non-coated iron oxide NPs showed unclear peaks at 2θ = 24.2, 33.2, 40.9, 49.5, 54.2, 65.2, and 62.5 corresponding to (012), (104), (113), (024), (116), (211), and (214) planes of iron oxide in the rhombohedral spinel phase which indexed in JCPDS card no. 01-084-0307. The appearance of iron oxide NPs instead of zerovalent iron indicates the amorphous nature of iron oxide NPs synthesized by BM extract as described in earlier literature (Jana et al., 2016). On the other hand, the ZnO-coated iron oxide NPs showed six distinctive peaks at 2 θ = 34.2, 35.3, 46.7, 54.7, 64.1, 66.2, and 72.2 which correspond to (002), (101), (102), (110), (200), (112), and (004) planes of hexagonal ZnO (JCPDS card no. 01-075-1533). In addition, four peaks at 2θ = 36.8, 41.4, 56.6, and 63.2, 72.2 correspond to (200), (210), (221), (311), and (004) planes of cubic ZnO (JCPDS card no. 01-077-2414). Another two weak peaks correspond to (220) and (400) planes of cubic magnetite (JCPDS card no. 01-086-1355), which prove the change in the crystal structure of iron oxide NPs after coating. Also, the absence of major peaks of iron oxide NPs indicates the integration of ZnO with iron oxide NPs (Yacaman et al., 2001; Phoohinkong et al., 2017; Jana et al., 2019).

**FIGURE 7:**
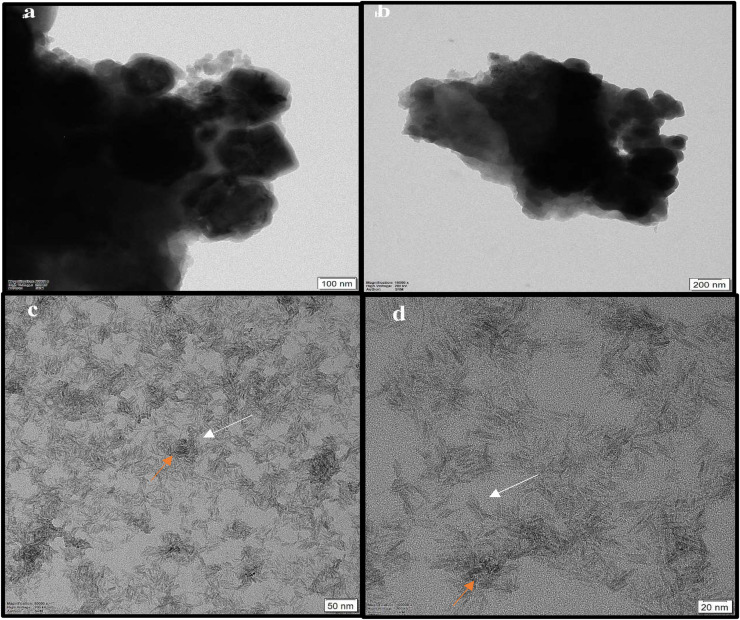
HRTEM micrograph of non-coated iron oxide NPs **(a,b)**, and iron oxide/ZnO nanocomposite **(c,d)**; orange arrows for iron oxide NPs and white arrows for ZnO NPs.

The HRTEM micrograph of non-coated iron oxide NPs represented aggregation of spherical and irregular shapes. However, the coating iron oxide NPs with ZnO NPs showed an aggregated rod shape of zinc oxide with approximately an average length of 19.25 ± 3.2 nm, and width 3.3 ± 0.6 nm surrounding amorphous iron oxide NPs (Figure 7). In that concern, Yacaman et al. (2001) explained that the identical energy of different configurations for NPs causes easily transformation of shapes (Jana et al., 2016). Till now, no data illustrate that the ZnO coating iron oxide NPs cause a change in the morphology or the crystal structure of iron oxide NPs. Moreover, Phoohinkong et al. (2017) recorded the size and morphological changes of copper-doped zinc oxide from rod-shaped (50–100 nm) to irregular NPs (5 nm) (Arakha et al., 2015). On the other hand, these results were different in the case of the synthesis of the αFe_2_O_3_/ZnO nanocomposite; the average size was five times as compared to α-Fe_2_O_3_ nanoparticles, and the shape was rod before and after coating (Abbas et al., 2020b).

**FIGURE 8:**
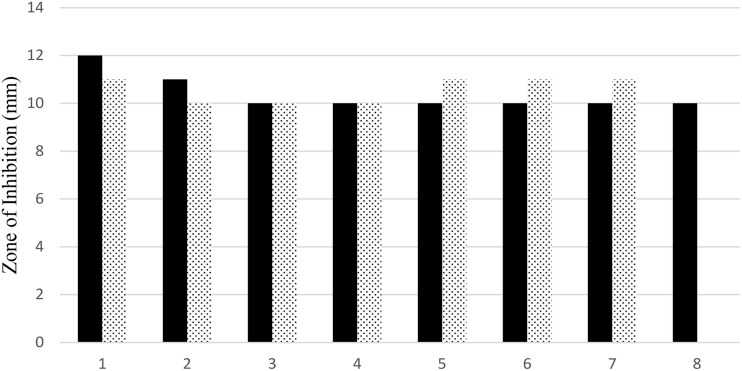
Antimicrobial efficiency of iron oxide NPs synthesized by different preparations of BM extract against both MRSA (black bar), and (dotted bar) *E. coli*. 1–4: 0.25, 0.5, 1, and 2 g of BM boiled in 100 ml water and 5–8: 0.25, 0.5, 1, and 2 g of BM soaked in 100 ml water.

**FIGURE 9:**
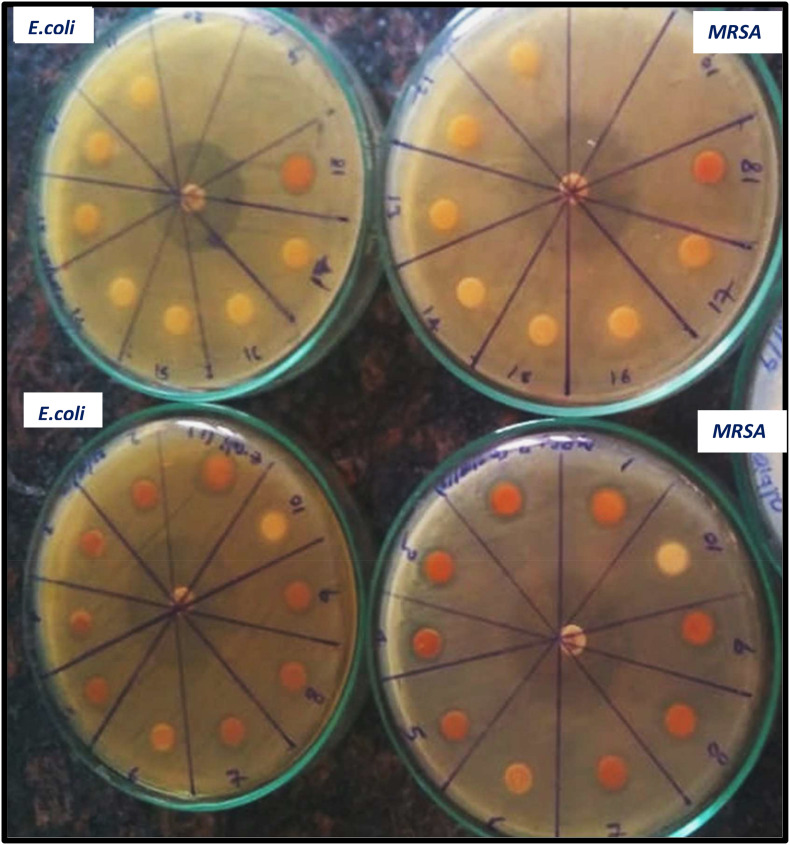
Antimicrobial efficiency of iron oxide NPs synthesized by different preparations of BM extract against both MRSA, and *E. coli* [Different preparations: 1–4: iron oxide NPs synthesized by BM boiled in 100 ml water and 5–9, 18: by BM soaked in 100 ml water, and 10, 11–17: negative control (plant extract without, iron NPs), positive control tetracycline (in the middle of the plate)].

The antimicrobial activity of both uncoated iron oxide NPs and iron oxide/zinc oxide nanocomposite has been examined using the agar disc diffusion method. The most perfect preparations of iron oxide NPs were those prepared by boiling 0.25 g of the plant in deionized water then the filtrate added to ferric chloride (1:1 v/v). Hence, the higher diameter of the inhibition zone was obtained against both MRSA and *E. coli* (Figures 8, 9). However, plant water-soaked extracts, plant water-boiled extracts, and ZnO-coated iron oxide NPs have no antimicrobial activity against pathogenic bacteria. The differences in the response of uncoated iron oxide NPs and iron oxide/zinc oxide nanocomposite toward pathogenic bacteria can be explained by their zeta potential where the high negative zeta potential for iron oxide NPs (−20.9 ± 6.24) and molecular crowding have antibacterial activity. However, the low negative zeta potential for iron oxide/zinc oxide nanocomposite prevents bacterial adherence. In that concern, recent studies have shown that nanoparticles’ zeta potential has a significant effect on bacterial adherence. Positively charged counterparts were expected to boost reactive oxygen species (ROS) production compared to negative charged and neutral nanoparticles. Negatively charged nanoparticles do not adhere to bacteria because of their negative potential. However, the molecular crowding of high concentration of negatively charged potential has a level of antibacterial action (Arakha et al., 2015; Abbas et al., 2020b).

## Conclusion

The progress of science and technology has created the pressing need of advancement green nanotechnology in biomedical research. Iron oxide NP biosynthesis and surface modification by other metal oxides, such as zinc oxide became important in therapeutic applications. The present paper focused on eco-friendly green methods for synthesis of iron oxide NPs and iron oxide/ZnO nanocomposite using the blepharis aqueous extract. The most stable colloidal iron oxide nanosuspension was prepared by using by boiling 0.25 g of the plant in deionized water then the filtrate added to ferric chloride (1:1 v/v). Iron oxide NP formation was demonstrated by UV spectroscopy in the absorption peak at 296 nm by the polyphenols of blepharis aqueous extract and redshift for the wavelength from 294 to 302 nm occurs after coating iron oxide NPs by ZnO NPs. In addition, the XRD analysis revealed the amorphous nature of non-coated iron oxide NPs, besides the integration of ZnO with iron oxide NPs after coating and the HRTEM for the coated iron oxide showed an aggregated rod shape of zinc oxide with an average length and width of 19.25 ± 3.2 nm × 3.3 ± 0.6 nm surrounding amorphous iron oxide NPs. Green iron oxide NPs had good antimicrobial activity against MRSA and *E. coli*. However, because of aggregation and the negative surface charge of the iron oxide nanocomposite, which causes the repulsion with the negative charge of bacteria surface, the iron oxide nanocomposite showed no antimicrobial activity. Future research on the cytotoxic effect of iron oxide NPs synthesized with polyphenols of BM extract will be suggested, and applications of such eco-friendly iron oxide NPs for bactericidal and medical purposes were recommended.

## Data Availability Statement

The raw data supporting the conclusions of this article will be made available by the authors, without undue reservation, to any qualified researcher.

## Author Contributions

HA and AK shared the idea and prepared nanoparticles, nanocomposite, and characterization. HA further writing, and shared MK antimicrobial activity.

## Conflict of Interest

The authors declare that the research was conducted in the absence of any commercial or financial relationships that could be construed as a potential conflict of interest.
